# Diverse Roles of F-BoxProtein3 in Regulation of Various Cellular Functions

**DOI:** 10.3389/fcell.2021.802204

**Published:** 2022-01-19

**Authors:** Zhiyang Zhang, Zhengqi Bao, Penglian Gao, Junyi Yao, Peter Wang, Damin Chai

**Affiliations:** ^1^ Department of Pathology, The First Affiliated Hospital of Bengbu Medical University, Bengbu, China; ^2^ Department of Orthopedics, The First Affiliated Hospital of Bengbu Medical University, Bengbu, China; ^3^ Bengbu Medical College Key Laboratory of Cancer Research and Clinical Laboratory Diagnosis, Bengbu Medical College, Bengbu, China

**Keywords:** Fbxo3, carcinomas, inflammation, rheumatoid arthritis, ubiquitination

## Abstract

Accumulated evidence shows that the F-box protein 3 (FBXO3) has multiple biological functions, including regulation of immune pathologies, neuropathic diseases and antiviral response. In this review article, we focus on the role of FBXO3 in inflammatory disorders and human malignancies. We also describe the substrates of FBXO3, which contribute to inflammatory disorders and cancers. We highlight that the high expression of FBXO3 is frequently observed in rheumatoid arthritis, leukemia, pituitary adenoma, and oral squamous cell carcinoma. Moreover, we discuss the regulation of FBXO3 by both carcinogens and cancer preventive agents. Our review provides a comprehensive understanding of the role of FBXO3 in various biological systems and elucidates how FBXO3 regulates substrate ubiquitination and degradation during various physiological and pathological processes. Therefore, FBXO3 can be a novel target in the treatment of human diseases including carcinomas.

## Introduction

Ubiquitination is one type of post-translational modifications (PTMs), which regulates cellular protein concentrations in eukaryotic organisms (Senft et al., 2018). The ubiquitin protease system selectively targets multiple proteins for degradation through the use of activating (E1), conjugating (E2) and ligating (E3) enzymes (Popovic et al., 2014). In particular, E3 ubiquitin ligases determine substrate specificity for ubiquitination and then transfer ubiquitin chains to the substrate, which leads to substrate degradation in the 26S proteasome (Grabbe et al., 2011). Ubiquitination is involved in the regulation of almost all cellular activities, including embryonic development, cell proliferation, apoptosis, autophagy, signal transduction and DNA repair (Grabbe et al., 2011; Popovic et al., 2014). In recent years, Bortezomib as a proteasome inhibitor has been approved for the treatment of multiple myeloma and mantle cell lymphoma (Palumbo et al., 2016). Targeting specific substrates for ubiquitination has become a new clinical therapeutic strategy.

The ubiquitin protein ligase complex Skp1-Cullin1-F-Box (SCF) is composed of four subunits: F-box protein, SKP1, CULLIN1, and RBX1 (Skaar et al., 2014). Twenty years after the discovery of the F-box protein family, around 70 different F-box proteins have now been identified in mammals (Skaar et al., 2013). F-box proteins are classified into three categories based on the type of C-terminal interaction: FBXW (containing WD40 repeats), FBXL (containing leucine-rich repeats), and FBXO (containing neither, but with other domains) (Wang et al., 2014). The F-box protein directs the SCF complex to specific substrates for ubiquitination. Emerging evidence has demonstrated that F-box proteins are associated with the aggressiveness of human tumors, cell cycle regulation, and regulation of the epithelial-mesenchymal transition (EMT) and cancer stem cells (CSCs) as well as drug resistance (Song et al., 2019; Yan et al., 2020). The F-box protein 3 (FBXO3), also known as FBX3, F-box only protein 3, F-box protein FBX3, and FBX, is encoded by a gene located on chromosome 11p13. Human FBXO3 gene has 13 splicing variants and belongs to the F-box protein family. Recently, studies have shown that FBXO3 participates in immune pathologies, neuropathic diseases, antiviral response, inflammatory disorders and human malignancies (Mallampalli et al., 2013; Lin et al., 2015; Shao et al., 2016; Hung et al., 2019). In the following paragraphs, we describe how FBXO3 contributes to inflammatory disorders and cancers, including leukemia, pituitary adenoma, oral squamous cell carcinoma and breast cancer. Moreover, we describe the regulatory mechanism of FBXO3 by carcinogens and cancer preventive agents.

### Structure of FBXO3

The human FBXO3 protein has 471 amino acids, with a molecular mass of 54,561 Da. Alternative splicing of FBXO3 gene generates two transcript variants diverging at the 3′ end. FBXO3 has two domains at its C-terminal: F-box domain (positions 10–56) and ApaG (Adenine tetraphosphate adenine G) domain (positions 278–408). ApaG domain is involved in mediating the ubiquitination and degradation of F-box and leucine-rich repeat protein 2 (FBXL2), resulting in cytokine gene transcription and promoting the progression of inflammation (Mallampalli et al., 2013). The relevant ApaG domain consists of an immunoglobin/fibronectin III-type fold and a classical β-sheet core. The central β-sheet core is a potential target in drug discovery, which aims at regulating inflammation and malignancies (Krzysiak et al., 2016). The FBXO3 has three described isoforms: isoform 1 (Q9UK99-1), isoform 2 (Q9UK99-2, 414–415: EM →VS, 416–471: Missing), and isoform 3 (Q9UK99-3, 36–40: Missing, 414–415: EM →VS, 416–471: Missing). The FBXO3 protein recognizes specific substrates for ubiquitination and degradation. Additional structural studies are needed to elucidate the functions of each domain of the FBXO3 protein in its various physiological and pathological processes.

### FBXO3 Regulates Inflammation

Following infection with a virulent pathogen, there is an excessive release of cytokines from proinflammatory cells, including macrophages, lymphocytes and polymorpho nuclear leukocytes (Dinarello, 2007; Sheu et al., 2010). This process, known as a “cytokine storm”, leads to hypercytokinemia where in hypercytokines it increases capillary permeability and tissue edema causing fever, pain, multiple organ failure, and even death (Nathan, 2002; Aird, 2003). TRAF proteins are cytokine signaling adapter proteins that are critically involved in inflammation and programmed cell death (Inoue et al., 2000; Mallampalli et al., 2013). FBXO3 proteins are critically involved in inflammation and target FBXL2 for degradation, partly promoting TNF receptor-associated factor (TRAF) signal transduction and cytokine gene transcription (Inoue et al., 2000; Mallampalli et al., 2013). Chen et al. reported that mice with FBXO3 knockout that are infected with *Pseudomonas aeruginosa* showed reduced lavage cytokine levels, protein concentrations, and proinflammatory cell counts in the lung tissue. This suggests that FBXO3 knockdown attenuates lung injury induced by *Pseudomonas* and reduces mortality (Chen et al., 2013). Typically, patients with sepsis die of organ dysfunction because of an unusually strong reaction response to an infection (Pantzaris et al., 2021). The researchers also found that subjects with sepsis had more TRAF and FBXO3 proteins and less FBXL2 protein in circulating white blood cells compared with control subjects. Moreover, the circulating FBXO3 and TRAF proteins in sepsis patients had positive correlations with cytokine responses (Chen et al., 2013).

Studies of the FBXO3 C-terminal structure demonstrate that the classical ApaG molecular features are indispensable for mediating FBXL2 ubiquitination and for promoting the release of cytokines (Krzysiak et al., 2016). This leads to the development of BC-1215 compound, which is a highly selective small molecule as a FBXO3 antagonist targeting the ApaG domain (Chen et al., 2013). BC-1215 decreases FBXO3-FBXL2 interaction in a dose-dependent manner and protects FBXL2 from FBXO3-induced degradation, which effectively lowers the expression of TRAF1-TRAF6 proteins. The addition of BC-1215 reduces proinflammatory cytokines and modestly inhibits bacterial growth in a mouse model of cecal ligation and perforation to induce sepsis (Chen et al., 2013). Treatment with BC-1215 or knockdown of FBXO3 were found to attenuate the inflammation of lung tissue induced by *Pseudomonas* and the H1N1 influenza virus, ear injury induced by tetradecanoylphorbol, and active colitis induced by acetate dextran sulfate sodium (Chen et al., 2013; Mallampalli et al., 2013). Furthermore, the downregulation of FBXO3 levels attenuates lung edema induced by ischemia-reperfusion (I/R) (Hung et al., 2019).

Oxygen glucose deprivation/re-oxygenation (OGD/R) model is often used for ischemia studies via oxygen and glucose deprivation and then reoxygenation to mimic ischemia/reperfusion injury condition. In the OGD/R model, miR-142-3p directly targets FBXO3 to ameliorate inflammation and apoptosis in SH-SY5Y cells (Li and Ma, 2020). The inflammasome is a complex composed of multiple proteins that regulate the maturation and release of pro-inflammatory cytokines, such as IL-1β and IL-18. Lipopolysaccharide (LPS) exposure attenuates FBXL2-induced NALP3 inflammasome ubiquitination by activating FBXO3, thereby increasing the secretion of IL-1β and IL-18 in inflammatory cells (Han et al., 2015). FBXO3 also potentiates vascular inflammation and increases atherosclerosis. Depletion of FBXO3 protein in macrophages eliminates oxidatively modified low-density lipoprotein-induced inflammation without affecting oxidized low-density lipoprotein uptake by macrophages (Chandra et al., 2019). Treatment with BC-1215 reduces the release of IL-1β and TNF-α (Chandra et al., 2019), which alleviates FBXO3-induced vascular inflammation and atherosclerosis. These data suggest that FBXO3 is a novel target of drug design that aims to alleviate atherosclerosis driven by pro-inflammatory cytokines. This benefit might extend beyond low-density lipoprotein reduction.

### FBXO3 Regulates Neuropathic Pain

Rab3-interactive molecule-1α (RIM1α) is essential for C-terminal regions-associated vesicle exocytosis (Schoch et al., 2006) and spinal plasticity in the presynaptic site of the dorsal horn, which contributes to the development of neuropathic pain. The voltage-gated N-type Ca^2+^ channel (Cav2.2) has been demonstrated that promoted neuropathic pain in mice model (Saegusa and Tanabe, 2014). Besides, it has been reported that RIM is related to Cav2.2 in neuropathic pain via promotion of vesicle exocytosis (Coppola et al., 2001; Hibino et al., 2002). One study demonstrated that FBXO3-dependent FBXL2 ubiquitination promotes RIM1α/CaV2.2 cascade in neuropathic pain based on spinal plasticity (Lin et al., 2015). FBXO3 degrades FBXL2 and leads to deubiquitination of RIM1, resulting in enhanced RIM1 interaction with the CaV2.2, which contributes to chronic pain due to upregulating CaV2.2 (Lai et al., 2016).

The roles of NcK-interacting kinase (TNIK) in neuropathic pain development is coupling TNIK–GluR1 and leads to subcellular redistribution of GluR1-AMPA receptors (AMPARs) (Hussain et al., 2010). TRAF2 enhances TNIK/GluR1 phosphorylation-dependent subcellular GluR1-AMPARs redistribution, leading to spinal nerve ligation-induced allodynia (Lin et al., 2015). Allodynia is a kind of pain due to a hypersensitive reaction to a normal stimulus. Similar to RIM1α, TRAF2 is also regulated by FBXO3-dependent FBXL2 (Chen et al., 2013; Mallampalli et al., 2013). FBXO3 is involved in neuropathic allodynia via its effects on degradation of FBXL2 and upregulation of TRAF2, and administration of BC-1215 ameliorates this allodynia (Lin et al., 2015). While the research in this field is still infancy, FBXO3 might provide a potential drug target for neuropathic pain relief.

### FBXO3 Regulates Autoimmunity Functions

Autoimmune regulator (AIRE) as a transcription factor is crucial for the maintenance of self-tolerance (Finnish-German, 1997; Nagamine et al., 1997). Impairment of AIRE activity is implicated in disturbed negative selection of T cells that are specific for self-antigens, which leads to lymphocytic infiltration of affected organs and causes disorders in immunological homeostasis. These ultimately result in autoimmune diseases, including type 1 diabetes mellitus, thymomas, and autoimmune thyroid diseases (Peterson and Peltonen, 2005; Sabater et al., 2005; Perheentupa, 2006; Gavanescu et al., 2007). FBXO3 has been reported to regulate autoimmunity by promoting the ubiquitination and transcriptional activity of the AIRE (Shao et al., 2016). AIRE is phosphorylated on the Thr-68 and Ser-156 residues near its N-terminus allowing it to bind to FBXO3 and becomes ubiquitinated. The ubiquitination of AIRE increases the activity of tissue-specific antigens (TSA) genes and enhances the recruitment of positive transcription elongation factor b (P-TEFb) to target genes (Shao et al., 2016), which influences the maturation of thymocytes (Peterson et al., 2008).

### FBXO3 Negatively Regulates Antiviral Response

Rift Valley fever virus (RVFV) infection can cause animal-derived diseases, which are transmitted to people mainly through mosquito bites or contact with infected livestock (Bird et al., 2009; Boshra et al., 2011). RVFV is prevalent in Africa and has caused frequent outbreaks that result in devastating loss of lives and properties. It is known that interferon activation is essential for antiviral response. One group showed that FBXO3 inhibits the antiviral response in host cells. As the main virulence factor of RVFV, the nonstructural protein NSs recruits FBXO3 to degrade the transcription factor TFIIH-p62 of host cells (Kainulainen et al., 2014). This facilitates the pathogenesis of RVFV by inhibiting transcriptional upregulation of the innate immunity and hindering the antiviral type I interferon (IFN-I) system to allow uncontrolled viral replication (Bouloy et al., 2001; Billecocq et al., 2004). NSs interacts with the full-length FBXO3 as well as with a truncated isoform that lacks the C-terminal acidic and poly(R)-rich domains (Kainulainen et al., 2014).

The genome structures of type 1 IFN in fish are similar with mammals (Kirsten et al., 2018). IRF3 and IRF7 are transcription factors of the IFN regulatory factor (IRF) family that induce IFN expression (Lu et al., 2019). IFN induces transcription of downstream antiviral genes through activation of JAK-STAT signaling pathway (Cheng et al., 2019). IRF3 and IRF7 degradation and IFN signaling activation are critical for FBXO3-mediated antiviral response in zebrafish. FBXO3 negatively regulates antiviral response by promoting K27-linked ubiquitination and proteasomal degradation of IRF3 and IRF7 in an F-box domain-independent manner (Li et al., 2020). In zebrafish model, FBXO3 deletion induces the expression of key antiviral genes and shows higher resistance to virus infection in liver and spleen (Li et al., 2020).

### Role of FBXO3 in Rheumatoid Arthritis

Rheumatoid arthritis (RA) is an autoimmune disease because immune system hurts healthy cells, leading to erosion of bone and cartilage in joints, especially in the hands, knees and wrists, which causes pain, stiffness, swelling and dysfunctions in the joints (Wang et al., 2019). To investigate the role of FBXO3 in RA, Masuda et al. examined the expression of a diverse array of genes through *in situ* hybridization (Masuda et al., 2002). They concluded that some proliferation-related molecules, including FBXO3, displayed higher expression levels in RA synovial tissues compared to normal synovial tissues (Masuda et al., 2002). The involvement of FBXO3 in RA has not been clearly demonstrated. It is known that F-box proteins regulate the cell cycle (Winston et al., 1999), FBXO3 might be potentially involved in survival and proliferation of RA synovial fibroblast (RA-SF).

### Role of FBXO3 in Amyotrophic Lateral Sclerosis

Amyotrophic lateral sclerosis (ALS) has a poor prognosis, and most of patients with ALS die within 3–7 years after involving the respiratory muscles (Talbott et al., 2016). The pathogenic mechanism is still unclear, and no effective treatment is available. Chromosome 21 open reading frame 2 (C21ORF2) interacts with never in mitosis gene A related kinase 1 (NEK1) that involve in DNA damage repair and regulate cell cycle, which is highly related to ALS development (Fang et al., 2015; Chia et al., 2018). Watanabe et al. showed that Fbxo3 targeted C21ORF2 for ubiquitination and degradation (Watanabe et al., 2020). Because NEK1 was stabilized by C21ORF2, depletion of FBXO3 stabilized both C21ORF2 and NEK1. NEK1-mediated phosphorylation of C21ORF2 can protect it from proteasome-dependent degradation due to attenuating the interaction between FBXO3 and C21ORF2 (Watanabe et al., 2020). Inhibition of NEK1 activity and increased degradation of C21ORF2 by FBXO3 may be potential approaches for treatment of patients with ALS.

### Role of FBXO3 in Human Cancers

#### FBXO3 Regulates Pathogenesis in Several Types of Cancers

FBXO3 has been demonstrated to participate in the occurrence and progression of a variety of human cancers. It is noteworthy that in acute promyelocytic leukemia, the PML gene is the site of the t (15, 17) chromosomal translocation wherein it is fused to the retinoic acid receptor (RAR) gene, leading to the generation of PML-RAR fusion protein (de Thé et al., 1990; de Thé et al., 1991; Goddard et al., 1991; Kakizuka et al., 1991). It has been shown that PML activates transcription by preventing FBXO3-catalyzed ubiquitination of HIPK and p300 (Shima et al., 2008). Conversely, PML-RAR enhances FBXO3-induced degradation of HIPK2 and p300 in a dose-dependent manner to inhibit transcription, which might contribute to the pathogenesis of leukemia (Shima et al., 2008). Another study by Laura et al. analyzed the relationship between FBXO3 and pituitary adenoma and showed that FBXO3 enhances the degradation of the aryl hydrocarbon receptor-interacting protein (AIP) through the rapid ubiquitin proteasome pathway, which has direct implications for the phenotype. Through this, a novel pathogenic mechanism of pituitary adenoma was generated (Hernández-Ramírez et al., 2016). In a work done by Cha et al., which employed a combination of array-based comparative genomic hybridization and multiplex ligation-dependent probe amplification, it was found that the highest deletion frequencies in FBXO3 might be related to the occurrence and progression of oral squamous cell carcinoma (Cha et al., 2011).

The FBXO3-ΔNp63α is critical for TGF-β-induced tumor metastasis (Hao et al., 2019). ΔNp63 has been identified as a subtype of p63 that leads to regulation of cell proliferation, cell adhesion, EMT, and inhibition of tumor metastasis (Bergholz and Xiao, 2012). FBXO3 promotes breast cancer metastasis through K48-linked polyubiquitination of the ΔNp63α. This process is independent of Smad but dependent of Erk (Niu et al., 2021). Upregulation of FBXO3 by activation of TGF-β results in the degradation of ΔNp63α with concomitant decreased expression of E-cadherin and desmoplakin (DPL) (Niu et al., 2021). In addition, the high expression of FBXO3 indicates poor prognosis in patients with breast cancer (Niu et al., 2021). Taken together, FBXO3 targets different substrates to participate in carcinogenesis.

### FBXO3 Regulates Smurf1 and BMP Pathway

The Smad ubiquitination regulatory factor 1 (Smurf1) is a member of the HECT-type E3 ubiquitin ligases and based on its C2-WW-HECT architecture, which belongs to the neural precursor cell-expressed and developmentally downregulated gene 4 (Nedd4) family of lipases (Fu et al., 2020; Wang et al., 2020). Smurf1 regulates several biological pathways, including the transforming growth factor beta (TGF-β), the bone morphogenetic protein (BMP), the non-canonical Wnt pathway, and the mitogen-activated protein kinase (MAPK) pathway (Fu et al., 2020). Smurf1 is also related to cell growth and migration, embryonic development, immune responses, and tumorigenesis. FBXO3 targets all the Nedd4 family members including Smurf1 for degradation (Li et al., 2015). FBXO3 upregulates BMP pathway by mediating Smurf1 ubiquitination *in vivo* as well as *in vitro* and FBXO3 significantly promotes the poly-ubiquitination of Smurf1 (Li et al., 2015).

### FBXO3 Regulates DNA Damage

The mouse homolog of diaphanous 2 (mDia2) belongs to the diaphanous-related formin 1 (mDia1) family. mDia2 influences the remodeling of actin and microtubule cytoskeletons after transformation to its active conformation (Chesarone et al., 2010), and plays a crucial role in cell invasion and cytokinesis (Lizárraga et al., 2009; Daou et al., 2014). FBXO3 forms a complex with mDia2 and p53, and co-expression of mDia2 and FBXO3 promotes p53-dependent apoptosis in an actin-nucleation-independent manner (Isogai et al., 2015). As a tumor suppressor, p53 regulates cell growth through cellular apoptotic programs and DNA repair. FBXO3 knockdown attenuates p53-mediated apoptosis upon DNA damage (Bieging and Attardi, 2012).

### FBXO3 Regulates Cell Apoptosis

Studies have shown that FBXO3 contributes to tumor progression but also increases tumor cell apoptosis. Recruitment of histone deacetylases by oncoproteins is a key inciting event for cancer progression (Hess-Stumpp, 2005; Minucci and Pelicci, 2006). In lung squamous cell carcinoma, Kong et al. demonstrated that treatment with the histone deacetylase inhibitor belinostat (PXD101) transcriptionally upregulates FBXO3 and FBXW10, which directly target son of sevenless (SOS), an upstream regulator of the MAPK pathway, to inhibit growth of tumor cells (Kong et al., 2017). Thus, the induction of tumor cell apoptosis is increased, and drug resistance to cisplatin is reduced. This suggests that targeting FBXO3 might be a novel strategy for cancer treatment.

### FBXO3 Is Regulates by miRNAs

MicroRNAs (miRNAs) are small non-coding RNAs that regulate target genes at the post-transcriptional level (Winkle et al., 2021). Notably, non-coding RNAs are essential for maintaining cellular homeostasis and perform their functions by regulating cell proliferation, migration, invasion, metastasis, and apoptosis (Goodall and Wickramasinghe, 2021; Jiang et al., 2020). The levels of some miRNAs have been shown to correlate with various cancers through negatively regulating genes including F-box proteins (Lin et al., 2019). Analyses using the TargetScan online computational algorithm (www.targetscan.org) and luciferase reporter genes showed that FBXO3 is identified as a target of miR-142-3p (Li and Ma, 2020). It has been shown that miR-142-3p plays key roles in tumorigenesis and cancer progression and is expressed at lower levels in breast tumor tissues than in those from normal individuals (Xu et al., 2020). miR-142-3p negatively regulates the canonical Wnt signaling pathway to regulate human breast cancer stem cells (Isobe et al., 2014). Several studies have also found that miR-142-3p is an important regulatory element in hepatocellular carcinoma, cervical cancer, non-small cell lung carcinoma, and glioblastoma (Chai et al., 2014; Xiao and Liu, 2015; Shrestha et al., 2017; Dong and Song, 2021). Furthermore, miR-142-3p has also been linked to inhibition of tumor progression and invasion. Thus, miR-142-3p might be useful in targeting cancer stem cells (Ghafouri-Fard et al., 2021). However, the relation between miR-142-3p to FBXO3 in human cancer cells is not yet clear and needs further research.

## Conclusion and Perspective

**FIGURE 1:**
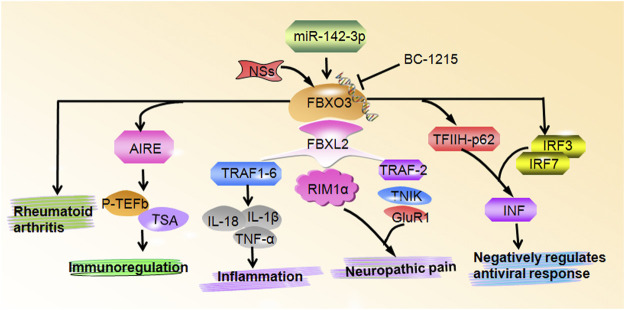
FBXO3 regulates multiple biological functions. FBXO3 controls numerous cellular signaling pathways and genes to participate in inflammation, neuropathic pain, immunoregulation, negatively regulates antiviral response and rheumatoid arthritis.

FBXO3 is involved in numerous biological functions and has an important impact on promoting inflammation, immune regulation, the inhibition of IFN-I that triggers virus replication, and the processes of neuropathic pain and rheumatoid arthritis (Table 1 and Figure 1). Among these, the most studies determine the role of FBXO3 in the pathophysiological mechanism of inflammation. According to previous researches, FBXO3 is a critical modulator of inflammation which can inhibit LPS stimulation of inflammatory responses by promoting and inhibiting the degradation of FBXL2 and TRAFs, respectively. A new area being explored in cancer research is the role of ubiquitination in inflammasome biology. Leucine-rich repeat receptors (NLRs) and melanoma 2 (AIM2)-like receptors (ALRs) families are important in the assembly of the inflammasome complex (Schroder and Tschopp, 2010). Among these, NLRP1, NLRP3, and NLRC4 are linked to inflammatory diseases and colorectal cancer (Jin et al., 2007; Man and Kanneganti, 2015). The formulation of new immunotherapy to regulate inflammasomes governed by ubiquitination can provide a novel strategy for the treatment of diseases.

**TABLE 1:** The roles of FBXO3 in various biological functions.

Functions	Targets	References
Regulates inflammation	FBXL2	Mallampalli et al. (2013)
Neuropathic pain	FBXL2	Lin et al. (2015)
Immunoregulation	AIRE	Shao et al. (2016)
Negatively regulates antiviral response	TFIIH-p62	Kainulainen et al. (2014)
Rheumatoid arthritis	N/A	Masuda et al. (2002)
Leukemia	HIPK, p300	Shima et al. (2008)
Pituitary adenoma	AIP	Hernández-Ramírez et al. (2016)
Oral squamous cell carcinoma	N/A	Cha et al. (2011)
Breast cancer	ΔNp63	Niu et al. (2021)
Tumorigenesis	Smurf1	Li et al. (2015)

**FIGURE 2:**
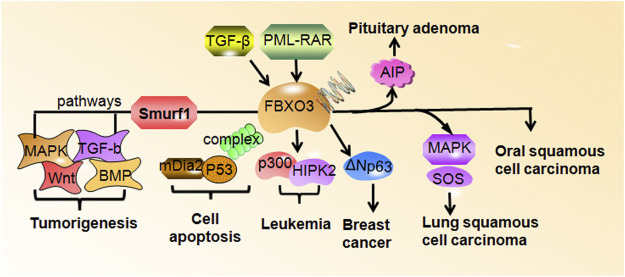
FBXO3 regulates downstream targets in human cancers. FBXO3 targets numerous genes to participate in tumorigenesis and cell apoptosis. FBXO3 is regulated by TGF-βand PML-RAR. The high expression of FBXO3 is frequent in rheumatoid arthritis, leukemia, pituitary adenoma, oral squamous cell carcinoma.

A review of literature shows contradicting reports on the effect of FBXO3 on tumor development (Figure 2). The expression of FBXO3 is increased in oral squamous cell carcinoma, acute promyelocytic leukemia, pituitary adenoma, and breast cancer. However, co-expression of FBXO3 and p53 promotes apoptosis of tumor cells (Bieging and Attardi, 2012). In addition, FBXO3 in combination with chemotherapeutic drugs can reduce drug resistance and increase chemical sensitization. These independent findings corroborate the potential roles of FBXO3 in the processes of anti-tumor activity and progression of tumors (Kong et al., 2017). While this may complicate the treatment, the specificity of FBXO3 makes it an attractive therapeutic target. However, the association of the expression of FBXO3 with tumor size, tumor stage, deep of infiltration, and prognosis in cancer patients has not yet been well established. The specific mechanism is also not clear, and there is still a lack of clinical data on FBXO3-related tumors.

In order to fully understand the role of FBXO3 in tumorigenesis, the following questions need to be addressed. What are the expression levels of FBXO3 in many human cancers other than oral squamous cell carcinoma, acute promyelocytic leukemia, and pituitary adenoma? What are the carcinogenic or anticancer signaling pathways that trigger FBXO3-induced oncogenesis? What are the targeted proteins of FBXO3 that are critically involved in tumor progression? What are other factors regulating the expression of FBXO3 in tumor cells? Is the high expression of FBXO3 associated with poor prognosis in various types of cancers? To find answers to these questions, FBXO3 knockout or knock-in transgenic mouse models could be used to validate the mechanism of FBXO3 in regulating the progression of human cancer. It is also important to look into various databases. Currently, the research on FBXO3 is still in its infancy, and further investigation is needed to develop better treatments using FBXO3 as a molecular target.
